# Phase II, randomized, open, controlled study of AS03-adjuvanted H5N1 pre-pandemic influenza vaccine in children aged 3 to 9 years: follow-up of safety and immunogenicity persistence at 24 months post-vaccination

**DOI:** 10.1111/irv.12295

**Published:** 2015-02-05

**Authors:** Javier Díez-Domingo, José-María Baldó, Maria Victoria Planelles-Catarino, María Garcés-Sánchez, Isabel Ubeda, Angels Jubert–Rosich, Josep Marès, Pilar Garcia-Corbeira, Philippe Moris, Maurice Teko, Carline Vanden Abeele, Paul Gillard

**Affiliations:** a FISABIO-Public HealthValencia, Spain; b Centro de Salud Quart de PobletValencia, Spain; c Centro de Salud PaiportaValencia, Spain; d Centro de Salud NazaretValencia, Spain; e Centro de Salud la ElianaValencia, Spain; f Centro de Salud MalvarrosaValencia, Spain; g Institut Pediàtric Marès RieraBlanes, Spain; h GSKMadrid, Spain; i GSK VaccinesWavre, Belgium; j ClinicsWaterloo, Belgium

**Keywords:** AS03-adjuvanted, cell-mediated immunity, children, H5N1 influenza vaccine, pre-pandemic

## Abstract

**Background:**

An AS03-adjuvanted H5N1 influenza vaccine elicited broad and persistent immune responses with an acceptable safety profile up to 6 months following the first vaccination in children aged 3–9 years.

**Methods:**

In this follow-up of the Phase II study, we report immunogenicity persistence and safety at 24 months post-vaccination in children aged 3–9 years. The randomized, open-label study assessed two doses of H5N1 A/Vietnam/1194/2004 influenza vaccine (1·9 μg or 3·75 μg hemagglutinin antigen) formulated with AS03_A_ or AS03_B_ (11·89 mg or 5·93 mg tocopherol, respectively). Control groups received seasonal trivalent influenza vaccine. Safety was assessed prospectively and included potential immune-mediated diseases (pIMDs). Immunogenicity was assessed by hemagglutination-inhibition assay 12 and 24 months after vaccination; cross-reactivity and cell-mediated responses were also assessed. (NCT00502593).

**Results:**

The safety population included 405 children. Over 24 months, five events fulfilled the criteria for pIMDs, of which four occurred in H5N1 vaccine recipients, including uveitis (*n* = 1) and autoimmune hepatitis (*n* = 1), which were considered to be vaccine-related. Overall, safety profiles of the vaccines were clinically acceptable. Humoral immune responses at 12 and 24 months were reduced versus those observed after the second dose of vaccine, although still within the range of those observed after the first dose. Persistence of cell-mediated immunity was strong, and CD4^+^ T cells with a T_H_1 profile were observed.

**Conclusions:**

Two doses of an AS03-adjuvanted H5N1 influenza vaccine in children showed low but persistent humoral immune responses and a strong persistence of cell-mediated immunity, with clinically acceptable safety profiles up to 24 months following first vaccination.

## Introduction

The evolution of avian influenza A/H5N1 strains with the potential to cause a human pandemic represents a serious threat to public health; thus, pandemic preparedness against A/H5N1 strains is paramount. Children are particularly vulnerable to morbidity and mortality associated with novel influenza A viruses because they lack previous exposure to influenza viruses and vaccines and, therefore, lack heterosubtypic immunity. In addition to the susceptibility to infection represented by immune naivety, viral transmission may be more extensive in children than adults because younger people shed viruses at high titers for long periods and have extensive social contact networks.^1^,^2^

Pandemic vaccines should provide antigen-sparing, cross-reactive immunogenicity allowing the use of prime-boost strategies and should be suitable for use in children because this group is important in viral transmission.^1^,^2^ Various prototype H5N1 influenza vaccines are licensed, which have been developed using antigens against prevalent avian influenza strains identified by global surveillance.^3^–^5^ One such vaccine is *Prepandrix*™ (GlaxoSmithKline [GSK] Vaccines), containing A/Indonesia/05/2005 formulated with an oil-in-water Adjuvant System (AS03), which has been shown to provide strong, durable, cross-clade immune responses in adults.^6^ In addition, we previously reported the results of a Phase II, randomized, open-label study in children aged 3–9 years and showed that two doses of H5N1 (A/Vietnam/1194/2004) vaccine containing 1·9 μg or 3·75 μg of hemagglutinin antigen (HA) adjuvanted with two different dosages of AS03, elicited strong antibody responses against the vaccine and drifted strains.^7^

In this study, we present the follow-up results of the Phase II, randomized, open study of AS03-adjuvanted H5N1 A/Vietnam/1194/2004 vaccine in children aged 3–9 years. We prospectively monitored safety for up to 24 months post-vaccination and assessed the persistence of immune responses at 12 and 24 months based on hemagglutination-inhibition (HI) antibody titers and cell-mediated immunity (CMI).

## Methods

This was a Phase II, open-label, randomized study conducted in seven centers in Spain. The inclusion criteria were as follows: healthy children aged 3–9 years at the time of first vaccination whose parents were able to comply with the protocol and provided written informed consent. The exclusion criteria list included: use of any investigational or non-registered product (drug or vaccine) within 30 days before study enrollment or planned use during the study; receipt of any chronic drug therapy with the exception of inhalative treatment for seasonal allergies or asthma; history of hypersensitivity or allergies to vaccines or vaccine components; administration of immunoglobulins or any blood products within 3 months before enrollment or planned during the study.

All protocols and study documentation were approved by an appropriate independent/local ethics committee(s) in accordance with Good Clinical Practice, the Declaration of Helsinki, and all regulatory requirements. (ClinicalTrials.gov NCT00502593). Parents/guardians provided informed written consent.

### Vaccines and randomization

The study vaccine was an H5N1 inactivated, split-virion recombinant influenza vaccine manufactured by GSK Vaccines (Dresden, Germany). The vaccine contained either 1·9 μg or 3·75 μg of HA of A/Vietnam/1194/2004 (H5N1) NIBRG-145 strain (National Institute for Biologic Standards and Control, Potters Bar, UK) and AS03 containing 11·86 mg (AS03_A_) or 5·93 mg (AS03_B_) tocopherol per dose.

The study was conducted in three stages. The children in Stage 1 received 1·9 μg HA with AS03_B_. Following a safety review by an Independent Data Monitoring Committee conducted 28 days after the first vaccination, children in Stage 2 received 3·75 μg HA with AS03_B_, and 14 days after the commencement of the second stage, those in Stage 3 received 3·75 μg HA with AS03_A_. Each stage also included a control group who received trivalent inactivated influenza vaccine (TIV; *Fluarix*™; GSK Vaccines, Belgium).

Children at each stage were randomized 3:1 to receive either the H5N1 vaccine or TIV using a central Internet-based randomization system and were allocated sequentially 1:1 to 6–9 years and 3–5 years age strata. The randomization list was generated by the sponsor, and randomization by center and age was performed using a minimization algorithm, in which each factor had equal weight. A summary of the study protocol can be accessed at http://www.gsk-clinicalstudyregister.com/ (GSK study ID 107066).

### Objectives

The co-primary objectives of the original study were to assess HI antibody responses against the vaccine strain (A/Vietnam/1194/2004) at days 21 and 42 post-vaccination and to assess solicited adverse events (AEs) 7 days post-vaccination and unsolicited AEs for 6 months post-vaccination. Exploratory analyses were performed to assess cell-mediated immunity (CMI) at 6 months post-vaccination in children aged 3–5 years in the 1·9 μg HA/AS03_B_ group.

In this follow-up report, we present the safety data up to 24 months post-vaccination in each vaccine group and the TIV control group, including AEs, serious AEs (SAEs), and AEs of special interest/ potential immune-mediated diseases (AESI/pIMDs). We also report HI antibody responses and CMI against the vaccine strain up to 24 months post-vaccination in the 1·9 μg HA/AS03_B_ (3–5 years) and 3·75 μg HA/AS03_A_ (3–5, 6–9 years) groups, as observations from these two groups were expected to represent the lowest and highest responses to H5N1 vaccination. HI antibody responses against vaccine homologous (A/Vietnam/1194/2004) and heterologous (A/Indonesia/5/2005) strains were assessed. The safety analysis and HI antibody responses were described as secondary outcomes in the protocol, and CMI as exploratory.

### Safety assessments

Blood samples were taken on days 0, 21, and 42 and at months 6, 12, and 24 for assessment of biochemistry safety parameters. Unsolicited AEs, SAEs, and AESI/pIMDs were assessed prospectively throughout the 24-month follow-up period and were graded for severity. Investigators provided assessment of the causal relationship between the study vaccines and AEs. All SAEs were to be reported within 24 hours. AEs were classified according to the Medical Dictionary for Regulatory Activities (MedDRA).

### Immunogenicity assessments

Blood samples were taken on days 0, 21, and 42 and at months 6, 12, and 24 for assessment of HI and CMI. All serological testing was performed in a central GSK Vaccines laboratory or a validated external laboratory using standardized, validated procedures. HI assays were performed using an established HI method, using horse rather than avian erythrocytes.^8^ All sera were tested in duplicate.

For intracellular cytokine staining, the method described by Maecker *et al*. was adapted,^9^ in which T cells were restimulated ex vivo by incubation with antigen in the presence of costimulatory antibodies to CD28 and CD49d^10^ and Brefeldin A to inhibit cytokine secretion and allow intracellular accumulation. The cells were then stained by fluorochrome-conjugated antibodies to phenotypic (CD4^+^ or CD8^+^) surface markers, intracellular cytokines, and activation marker before enumeration by flow cytometry. In addition to influenza H5N1 split antigen, H5 peptide pools (with overlaps to ensure all T-cell epitopes are represented) were used as ex vivo stimuli to specifically measure the response to H5 antigen. Antibodies used for cell stimulation were unconjugated and azide-free anti-CD28 and anti-CD49d. Conjugated antibodies used for staining were anti-IFN-γ-FITC, anti-IL-2-allophycocyanin, anti-TNF-α-PE Cy7, anti-CD40L-PE, anti-CD4-PerCP, and anti-CD8-allophycocyanin Cy7 (BD Biosciences, San Jose, CA, USA).

Purified peripheral blood mononucleated cells (PBMCs) were thawed, washed twice in culture medium [RPMI 1640 (Cambrex, Charles City, NJ, USA)] supplemented with 10% heat-inactivated FCS (PAA Laboratories GMbH, Pasching, Austria), 10000 IU/ml penicillin, 10000 μg streptomycin sulfate, 200 mm L-glutamine, 100X MEM nonessential amino acids, 100 mm sodium pyruvate, 50 mm 2-mercapto-ethanol (all from Life Technologies, Gent, Belgium), examined for viability and counted (*Trucount*, BD Biosciences), washed again and resuspended to 2 × 10^7^ cells/ml in culture medium. The PBMCs (10^6^ cells per well) were incubated in 96-well microtiter plates with costimulatory anti-human CD28 and CD49d antibodies (1/250 dilution each) and stimulated for 20 h at 37°C with either H5N1 split antigen from the A/Vietnam/1194/2004 NIBRG-14 vaccine strain (final concentration 1 μg/ml HA) or one of the peptide pools (final concentration 1·25 μg/ml of each peptide). Brefeldin A (BD Biosciences) at a final concentration of 1 μg/ml was added for the last 18 h of culture. An unstimulated control (no antigen) and positive control (staphylococcus enterotoxin B, 1 μg/ml; Sigma-Aldrich) were included in each assay. Following incubation, the cells were washed (phosphate-buffered saline containing 1% FCS) and stained with anti-CD4-PerCP and anti-CD8-APC Cy7. The cells were then washed again, fixed and permeabilized with the Cytofix/Cytoperm kit (BD Biosciences) according to instructions, and stained with anti-IFNγ-FITC, anti-IL-2-APC, anti-TNF-α-PE Cy7, and anti-CD40L-PE. Following washing (Perm/Wash buffer, BD Biosciences), the cells were analyzed by flow cytometry.

Cells were analyzed on a FACSCanto flow cytometer (BD Biosciences) using 6 color panels. Data were analyzed using *FACSDiva* software (BD Biosciences). Results were expressed as frequencies of CD4^+^ or CD8^+^ T cells responding to the antigen and expressing at least two of the markers (CD40L, IFN-γ, IL-2, TNF-α) per million total CD4^+^ or CD8^+^ T cells. Background (unstimulated control) was subtracted from all values. The remaining positive events were regarded as significant. Samples were only included for analysis if viability was 80% or more.

### Analyses

The overall target sample size was 400 participants, including 100 participants in each stage to receive H5N1 vaccine formulations and 100 participants to receive TIV distributed evenly among the 3 stages. The sample size calculation was based on the primary endpoint as previously described.^7^

The safety analyses were performed on the total vaccinated cohort (TVC), including children who received ≥1 dose of vaccine. Safety data were analyzed descriptively with a 95% confidence interval (CI).

Analyses of HI and CMI were performed on the per-protocol immunogenicity persistence cohort including children in the TVC with no exclusion criteria, no protocol deviations, and with immunogenicity data available at a given time-point from 12 to 24 months. HI responses were analyzed descriptively based on the antilog of the arithmetic mean of the log-10 transformed titers (Geometric Mean Titer; GMTs), seroconversion rate (SCR; proportion of children with pre- and post-vaccination titers of >1:10 and ≥1:40, respectively, or pre-vaccination titers of ≥1:10 and a ≥ 4-fold post-vaccination increase in titer), seroprotection rate (SPR; proportion of subjects with post-vaccination titer ≥1:40). HI was tabulated with 95% CI. Children were considered seropositive if they had a pre-vaccination antibody titer of ≥1:10. For the CMI analysis, the results were expressed as the frequency of CD4^+^ and CD8^+^ T cells per million CD4+ T cells presented as median values with lower and upper quartiles.

## Results

**Figure 1:**
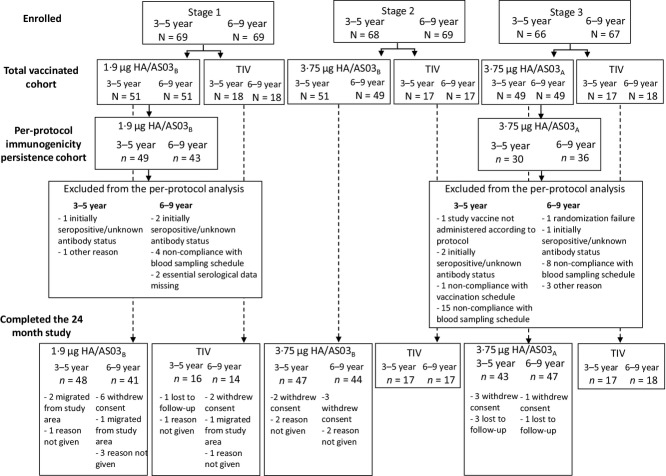
Participant flow. TIV, inactivated trivalent influenza vaccine; AS03, tocopherol-based oil-in-water Adjuvant System; A, 11·86 mg tocopherol; B, 5·93 mg tocopherol; HA, hemagglutinin antigen; N, number of subjects in group (total vaccinated cohort); *n*, number of subjects with data available (per protocol cohort).

A total of 408 children were enrolled including 405 children in the TVC (Figure1). Stage 1 (1·9 μg HA/AS03_B_) started on July 23, 2007, and the final contact (Month 24) was on December 4, 2009; Stage 2 (3·75 μg HA/AS03_B_) started on November 7, 2007, and the final contact was on February 9, 2010; Stage 3 (3·75 μg HA/AS03_A_) started on November 21, 2007, and the final contact was on April 14, 2010. In stages 1, 2, and 3, among children who received H5N1 vaccine, 89, 91, and 90 children, respectively, completed the 24-month study. Demographic details for all children in the TVC (safety analyses) have been previously reported.^7^ Demographic details for children in the per-protocol immunogenicity persistence cohort are shown in Table1.

**Table 1:** Baseline characteristics of vaccinated children (per-protocol immunogenicity persistence cohort)

	1·9 μg HA/AS03_B_		3·75 μg HA/AS03_A_	
	3–5 yrs *n* = 49	6–9 yrs *n* = 43	3–5 yrs *n* = 30	6–9 yrs *n* = 36
Age, years				
Mean ± SD	3·9 ± 0·83	7·6 ± 1·07	4·2 ± 0·94	7·0 ± 1·01
Median	4·0	8·0	5·0	7·0
Range (min–max)	3–5	6–9	3–5	6–9
Sex, *n* (%)				
Male	26 (53·1)	22 (51·2)	17 (56·7)	23 (63·9)
Female	23 (46·9)	21 (48·8)	13 (43·3)	13 (36·1)
Race and ethnicity, *n* (%)				
White – Caucasian/European heritage	47 (95·9)	42 (97·7)	29 (96·7)	36 (100)
Other	2 (4·1)	1 (2·3)	1 (3·3)	0

HA, hemagglutinin antigen; AS03, oil-in-water Adjuvant System containing 5·93 mg or 11·86 mg tocopherol (AS03_B_ and AS03_A_, respectively); n, number of subjects with data available (per protocol cohort); SD, standard deviation.

### Safety

#### AESI/pIMDs

Over the 2-year follow-up, five unsolicited AEs fulfilled the definition of AESI/pIMD. There was 1 case of vitiligo in each group in Stage 1: 1·9 μg HA/AS03_B_ (3–5 years) and TIV (6–9 years). Each case of vitiligo was medically attended, classed as mild in severity, and not considered to be related to vaccination. In Stage 2, there was one case of grade 2 autoimmune hepatitis in the 3·75 μg HA/AS03_B_ group (3–5 years) and 1 case of Type 1 diabetes mellitus in the TIV group (3–5 years). The case of autoimmune hepatitis was diagnosed 294 days after the first dose in a child who had increased transaminases at baseline; this pIMD was considered to be study vaccine related and was reported to be “recovering/resolving” at 24 months. The case of Type 1 diabetes mellitus was reported 132 days after the first dose, required hospitalization, and was not considered to be vaccine-related. During Stage 3, there was 1 case of grade 3 uveitis reported 8 days after the first dose of 3·75 μg HA/AS03_A_ (3–5 years), which was medically attended and considered to be vaccine-related; the child was reported to be recovered 51 days after the first dose.

#### Unsolicited AEs and SAEs

An overview of AEs reported during the 24-month study is shown in Table2. There were no apparent differences in the frequency or severity of AEs between the H5N1 vaccine and TIV groups or between the 3–5 years and 6–9 years age strata. In Stage 1, there were no SAEs reported during the 24-month study. In Stage 2, the case of autoimmune hepatitis (3·75 μg HA/AS03_B_) and the case of Type 1 diabetes (TIV) were reported as SAEs (described above as AESIs/pIMDs); there were no other SAEs during this stage. In Stage 3, there were three SAEs in 3·75 μg HA/AS03_A_ recipients, which were gastroenteritis (3–5 years), traumatic brain injury (3–5 years), and wound (6–9 years); these SAEs were not considered by the investigator to be vaccine-related. Withdrawals during the initial 6 months are previously reported.^7^ There were no withdrawals for AEs between 6 and 24 months.

**Table 2:** Number of subjects with adverse events by vaccine group and time after vaccination in the total vaccinated cohort

	Vaccine	Age, years	21 days after first vaccination and 30 days after second vaccination	Day 51 to 6 months	6 months to 24 months
Stage 1	1·9 μg HA/AS03_B_	3–5 (N = 51)	29	1	1
		6–9 (N = 51)	10	0	0
	TIV	3–5 (N = 18)	12	1	0
		6–9 (N = 18)	5	0	1
Stage 2	3·75 μg HA/AS03_B_	3–5 (N = 51)	28	0	1
		6–9 (N = 49)	19	1	0
	TIV	3–5 (N = 17)	9	2	0
		6–9 (N = 17)	6	0	0
Stage 3	3·75 μg HA/AS03_A_	3–5 (N = 49)	26	1	0
		6–9 (N = 49)	27	1	1
	TIV	3–5 (N = 17)	8	0	0
		6–9 (N = 18)	6	1	0

HA, hemagglutinin antigen; AS03, oil-in-water Adjuvant system containing 5·93 mg or 11·86 mg tocopherol (AS03_B_ and AS03_A_, respectively); TIV, trivalent inactivated influenza vaccine; N, number of subjects in group (total vaccinated cohort).

### Immunogenicity

#### Vaccine homologous HI antibody responses

**Figure 2:**
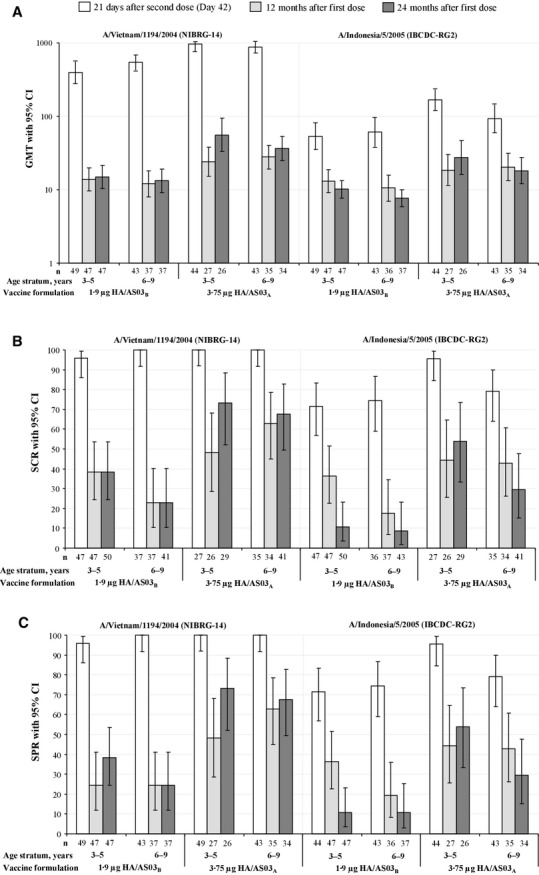
Immunogenicity in the per-protocol immunogenicity cohort (Day 42)^†^ and the per-protocol persistence cohort (months 12 and 24). HA, hemagglutinin antigen; AS03, oil-in-water Adjuvant System containing 5·93 mg or 11·86 mg tocopherol (AS03_B_ and AS03_A_, respectively); *n*, number of subjects with data available (per protocol cohort); CI, confidence limit; GMT, geometric mean titer; SCR, seroconversion rate (proportion of children with pre- and post-vaccination titers of >1:10 and ≥1:40, respectively, or pre-vaccination titers of ≥1:10 and ≥4-fold post-vaccination increase in titer); SPR, seroprotection rate (proportion of subjects with post-vaccination titer ≥1:40). ^†^Day 42 results show the per-protocol immunogenicity cohort from the original study which has been previously published^7^.

HI against A/Vietnam by age strata is shown (Figure2). At Month 12, overall (both age strata combined), 44·0% (95% CI: 33·2–55·3) of the children in the 1·9 μg HA/AS03_B_ group and 74·2% (95% CI: 61·5–84·5) of the children in the 3·75 μg HA/AS03_A_ group were seropositive for A/Vietnam antibodies (titer ≥1:10). At Month 24, overall, 48·8% (95% CI: 37·7–60·0) and 83·3% (95% CI: 71·5–91·7) were seropositive. Overall, GMTs 21 days after the second vaccination (Day 42) were 420·2 (95% CI: 333·5–529·4) in the 1·9 μg HA/AS03_B_ group and 969·0 (95% CI: 824·8–1138·4) in the 3·75 μg HA/AS03_A_ group, and at Month 12 were 13·1 (95% CI: 10·0–17·0) in the 1·9 μg HA/AS03_B_ group and 26·0 (95% CI: 19·6–34·5) in the 3·75 μg HA/AS03_A_ group. Responses at Month 24 were in the same range as those observed after the first vaccination at Day 21 and remained above baseline values. The SPRs at months 12 and 24 versus Day 42 were reduced, and the observed values were higher in the 3·75 μg HA/AS03_A_ group than the 1·9 μg HA/AS03_B_ group. At both months 12 and 24, the observed SCRs were higher in the 3·75 μg HA/AS03_A_ group than the 1·9 μg HA/AS03_B_ group. There were no marked differences between the age strata in the immune responses against A/Vietnam for any of the immunogenicity parameters.

#### Vaccine heterologous HI antibody responses

HI against containing A/Indonesia/05/2005 by age strata is shown (Figure2). At months 12 and 24 overall (both age strata combined), respectively, 39·8% (95% CI: 29·2–51·1) and 35·7% (95% CI: 25·6–46·9) of children in the 1·9 μg HA/AS03_B_ group, and 61·3% (95% CI: 48·1–73·4) and 66·7 (95% CI: 53·3–78·3) of the children in the 3·75 μg HA/AS03_A_ group, were seropositive for A/Indonesia antibodies. Overall, GMTs at months 12 and 24 were 11·9 (95% CI: 9·1–15·5) and 9·0 (95% CI: 7·4–10·9), respectively, in the 1·9 μg HA/AS03_B_ group, and 19·6 (95% CI: 14·4–26·6) and 21·8 (95% CI: 15·8–30·1), respectively, in the 3·75 μg HA/AS03_A_ group. At Month 24, overall, the SPR and SCR against A/Indonesia were 10·7% (95% CI: 5·0–19·4) and 9·8% (95% CI: 4·3–18·3), respectively, in the 1·9 μg HA/AS03_B_ group, and 40·0% (95% CI: 27·6–53·5) and 40·0% (95% CI: 27·6–53·5), respectively, in the 3·75 μg HA/AS03_A_ group.

#### CMI responses

**Figure 3:**
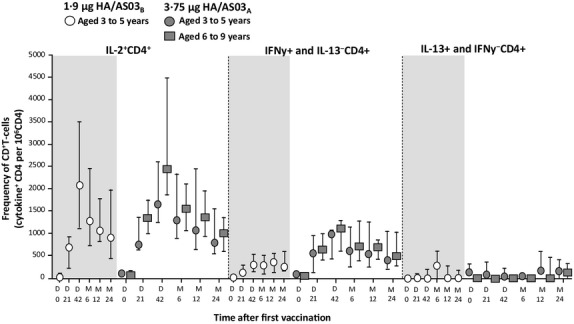
Cell-mediated immune responses against H5N1 vaccines in children from the per-protocol cohort for persistence. HA, hemagglutinin antigen; AS03, oil-in-water Adjuvant system containing 5·93 mg or 11·86 mg tocopherol (AS03_B_ and AS03_A_, respectively); 1·9 μg HA/AS03_B_: *n* = 29 (D0, M12), *n* = 26 (D21), *n* = 27 (D42), *n* = 23 (M6), *n* = 25 (M24) 3·75 μg HA/AS03_A_: 3–5 years: *n* = 11 (D0, D42, M24), *n* = 10 (D21), and *n* = 15 (M6, M12); 6–9 years: *n* = 16 (D0, D42), *n* = 17 (D21), *n* = 14 (M6), *n* = 12 (M12), and *n* = 15 (M24). Values are expressed as medians with first and third quartiles for days (D) 0, 21, and 42 and months (M) 6, 12, and 24.

**Figure 4:**
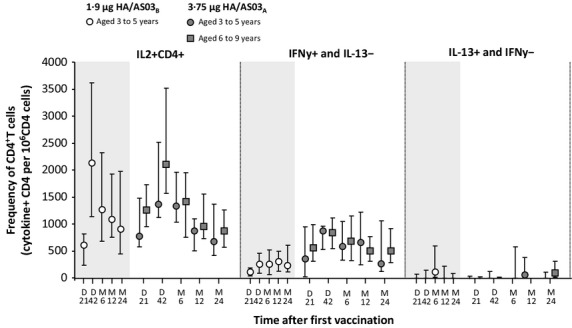
Cell-mediated immune responses against pool of peptides spanning the whole A/Vietnam/1194/2004 HA sequence in children from the per-protocol cohort for persistence. HA, hemagglutinin antigen; AS03, oil-in-water Adjuvant system containing 5·93 mg or 11·86 mg tocopherol (AS03_B_ and AS03_A_, respectively); 1·9 μg HA/AS03_B_: *n* = 26 (D21), *n* = 24 (D42), *n* = 6 (M6), *n* = 24 (M24) 3·75 μg HA/AS03_A_: 3–5 years: *n* = 8 (D0), *n* = 9 (D21), *n* = 10 (M6, M12), *n* = 7 (M24); 6–9 years: *n* = 16 (D21), *n* = 14 (D42), *n* = 13 (M6), *n* = 10 (M12), and *n* = 14 (M24). Values are expressed as medians with first and third quartiles for days (D) 21, and 42 and months (M) 6, 12, and 24.

In line with the preliminary findings reported previously,^7^ the frequency of CD4^+^ T cells producing IL-2 increased at Day 21 in both groups with a marked increase to a peak on Day 42 (Figure3). The magnitudes of the specific response to the H5N1 split antigen then decreased slightly until Month 24, where the frequency of CD4^+^ T cells producing IL-2 remained at a level markedly above pre-vaccination levels. The frequency of H5N1-specific CD4^+^ T cells identified as producing IL-2 and IFNγ (but not IL-13) was high in both groups. No effect of vaccination on the frequency of cytokine-positive CD8^+^ T cells was apparent. A similar pattern of CD4^+^ T-cell responses were also observed after stimulation with a pool of peptides spanning the whole A/Vietnam/1194/2004 HA sequence, but responses were less pronounced than with the H5N1 split antigen (Figure4). In line with the preliminary findings reported previously,^7^ the magnitudes of long-term CMI responses to the H5N1 split antigen and the pool of peptides continued to be markedly higher in the adjuvanted vaccine groups compared with the TIV control groups.

## Discussion

In this long-term follow-up study of a Phase II, randomized open-label study of AS03-adjuvanted H5N1 pandemic vaccine in children aged 3–9 years, we showed that antibodies against the vaccine strain persisted for up to 24 months after vaccination and that the long-term safety profile was acceptable.

In the first paper from this study, we showed that AS03-adjuvanted H5N1 vaccine was associated with a higher rate of injection site reactions than control (TIV) vaccine during the 7-day post-vaccination period, and up to 6 months post-vaccination, there were no SAEs in children who received 1·9 μg HA/AS03_B_, 1 SAE resulting in withdrawal (autoimmune hepatitis) in the 3·75 μg HA/AS03_B_ group, and 3 SAEs in the 3·75 μg HA/AS03_A_ group.^7^ In this long-term follow-up, there were no SAEs reported between 6 and 24 months, and over the entire 2 years, the severity and frequency of AEs in the H5N1 vaccine groups were consistent with those observed in the TIV control groups. In addition to prospectively recording AEs, in line with the regulatory recommendations, we monitored neurological and autoimmune events and assessed our safety data to identify AESI/pIMDs based on MedDRA Preferred Terms.^11^ Over the 24 month follow-up, we identified 5 events that fulfilled the criteria for AESIs/pIMDs, of which a case of autoimmune hepatitis and a case of uveitis were considered by the investigators to be vaccine-related, and which occurred during the first 6 months, as previously discussed.^7^

To date, this is the first study of AS03-adjuvanted H5N1 vaccine in children to assess safety up to 24 months after vaccination, and our results suggest that the long-term safety profile of the vaccine is clinically acceptable. Notably, the child with autoimmune hepatitis had increased concentrations of serum transaminases at baseline, and the child with uveitis also experienced tonsillitis, gingivostomatitis, and fever one day after the second dose of vaccine and seven days before the development of uveitis, raising the possibility of a viral infection as the cause of the subject's symptoms. Although there are no other long-term studies of AS03-H5N1 vaccines in children, mass vaccination during the 2009 swine-origin pandemic outbreak provided important safety data regarding AS03-adjuvanted A/H1N1pdm09 vaccine. Recent reports have suggested a link between an A/H1N1pdm09 vaccine (a strain distinct from the one we used)^12^ or natural infection^13^ with subsequent onset of narcolepsy. Further research will help to elucidate the chain of events that resulted in narcolepsy and the potential roles of genetic and environmental factors in triggering this disease. No cases of narcolepsy were detected in this H5N1 study, which was likely too small to detect rare events such as narcolepsy.

Our immunogenicity analysis showed that there was a low level of persistence of the HI antibody responses up to 24 months following the first vaccination. In addition to the original study, the results of this long-term follow-up suggest that AS03-H5N1 vaccine provides antigen-sparing, durable, cross-reactive immune responses in children, which will allow the use of prime-boost strategies.^7^

In addition to the humoral immune response, CD4+ T cells play an important role in immunity to influenza and are reported to have protective roles that are independent of neutralizing antibody responses.^14^,^15^ Various mechanisms underlying cell-mediated immunity to influenza have been reported, including the induction of antigen-specific T and B cells associated with long-term immune memory, characterized by influenza-specific human CD4+ T cell memory responses that are strongly biased toward T_H_1 cells, producing TNFα, IFNγ, and IL-2 on activation. The induction of immune memory via mechanisms that are independent of neutralizing antibody responses may be an important facet of vaccine protection against pandemic influenza because memory CD4+ T cells have been implicated in heterotypic immunity in animal models and in human challenge studies with H3N2 and H1N1 viruses.^16^–^19^

A previous study has shown that formulation of H5N1 vaccine with AS03 enhances B-cell memory and CD4+ T cells compared with non-adjuvanted vaccine in adults,^20^ and although the exact function of CD4+ T cells induced by AS03-H5N1 vaccine is not known, pre-clinical models suggest that α-tocopherol modulates the innate immune response and enhances adaptive immunity.^21^ To assess the induction of T-cell responses in children, we evaluated intracellular cytokines against H5N1 vaccines and a H5 pool of peptides. In the initial report from this study, in children aged 3–5 years receiving the 1·9 μg HA/AS03_B_ formulation or TIV, the CD4^+^ T-cell response to the H5N1 antigen was substantially induced upon vaccination with the AS03-adjuvanted H5N1 influenza vaccine, but not TIV, thus suggesting that AS03 enhances cell-mediated immunity.^7^ The CD4+ T-cell response was characterized by the production of IL-2 and IFN-γ, but not IL-13.^7^ In this follow-up study, the frequencies of specific CD4^+^ T cells peaked at Day 42 and remained markedly above pre-vaccination levels by Month 24, induced by the vaccine antigen and the peptide pool. The frequencies of vaccine-induced antigen-specific CD4^+^ T cells were identified as producing IL-2 upon short-term *in vitro* stimulation with the H5N1 split antigen, and a substantial portion of CD4^+^ T cells were IFNγ^+^ and IL-13^−^. However, CD8^+^ T-cell responses were not present, or not detectable, in the periphery at the analyzed time-points. Although further studies are needed to assess the mechanisms of cell-mediated immunity with influenza vaccines, our results suggest that AS03-H5N1 vaccine may induce CD4+ T-cell responses that may be associated with long-term, heterosubtypic immune memory in children.

In conclusion, up to 24 months following the first vaccination in children aged 3–9 years, AS03-adjuvanted H5N1 prepandemic vaccine demonstrated a clinically acceptable safety profile. There was a progressive reduction of humoral immunity at 12 and 24 months following the first vaccination, and strong, long-term, cross-reactive cell-mediated immune responses.

## Trademark statement

*Prepandrix*™ and *Fluarix*™ are trademarks of the GSK group of companies.
